# Editorial: Bioaerosol emission characteristics and the epidemiological, occupational, and public health risk assessment of waste and wastewater management

**DOI:** 10.3389/fpubh.2023.1111457

**Published:** 2023-02-17

**Authors:** Cheng Yan, Carla Viegas, Yunping Han, Annalaura Carducci

**Affiliations:** ^1^School of Environmental Studies, China University of Geosciences, Wuhan, China; ^2^H&TRC - Health & Technology Research Center, ESTeSL - Escola Superior de Tecnologia da Saúde, Instituto Politécnico de Lisboa, Lisbon, Portugal; ^3^NOVA National School of Public Health, Public Health Research Centre, Comprehensive Health Research Center, CHRC, NOVA University Lisbon, Lisbon, Portugal; ^4^Comprehensive Health Research Center (CHRC), Lisbon, Portugal; ^5^State Key Laboratory of Environmental Aquatic Chemistry, Research Center for Eco-Environmental Sciences, Chinese Academy of Sciences, Beijing, China; ^6^University of Chinese Academy of Sciences, Beijing, China; ^7^Laboratory of Hygiene and Environmental Virology, Department of Biology, University of Pisa, Pisa, Italy

**Keywords:** wastewater treatment, bioaerosol, source apportionment, emission release principle, occupational and public health risk assessment

## Why is important to assess occupational exposure to bioaerosols

Waste management industries are vital to achieve the Sustainable Development Goals (SDGs) suggested by World Health Organization (1). Besides SDGs achievement, also the circular economy is dependent of the waste sector. The circular economy intends to re-use the existing resources instead of disposing materials that are no longer useful (2). Thus, the circular economy is very dependent of an increased workforce dedicated to waste management. Nonetheless, while better waste management is being predicted as a critical contribute to reduce health outcomes and environmental negative impacts, the bioaerosols' occupational exposure in waste sector is being neglected leading to negative outcomes on workers' health (1).

The scientific community and stakeholders should be also aware of the foreseen increased exposure to microbes and antimicrobial resistance in different environments that will lead to a boost of exposure to bioaerosols (for instance mycotoxins and climate-sensitive infectious diseases) in the waste sector, mainly due to climate change (3, 4).

## Overview of the published papers

Aiming to assess health risks due to bioaerosol emission and air pollution, four articles contributed with their results. Kontro et al. found that bioaerosol levels were high especially in the composting compared to bioenergy producing facilities. Endotoxin, *A. fumigatus* and *Streptomyces* spp. detected in bioaerosols were also observed in the nasal passages of the workers, indicating that bioaerosols in composting plant has great potential to harm to workers' health.

Pascale et al. revealed high contamination levels and large microbial heterogeneity both for PM and bioaerosol samples. They also found *Bacillus* spp., *Saccharopolyspora* spp., and *Thermomyces* spp. may be suggested as indicators of biological contamination in composting plants. In addition, this work showed that using multiple assays is helpful to ensure the needed sensitivity and accuracy of bioaerosol detection.

Pan et al. assessed airborne bacterial community in electronic waste dismantling site and a waste transfer station based on culture-dependent and culture-independent methods. Bacterial communities in waste-associated bioaerosols were predominated by *Proteobacteria* spp. and *Bacteroidetes* spp.. One-third of the species in these genera were uncultured. Differences community structure existed in airborne bacterial diversity among different sampling sites, showing that waste-associated environments have unique bacterial diversity.

Bai et al. used the Taiwan Longitudinal Health Insurance Database and the Taiwan Air Quality Monitoring Database to conduct a retrospective cohort study to investigate whether air pollution increases the risk of uveitis. Overall, 175,489 subjects were linked to their nearby air quality monitoring stations; air pollution was significantly associated with incidental uveitis, especially at high total hydrocarbon (THC) and CH_4_ levels; and uveitis risk increased with increasing NO_x_ and THC levels.

For public health risk assessment of wastewater, Lin et al. reviewed the effects of water pollution on human health and disease heterogeneity. Eighty-five relevant papers were selected. The results shown that the impact of water pollution on human health is significant, although there may be regional, age, gender, and other differences in degree. Taken together, diarrhea caused by enteroviruses in aquatic environments is the most common disease caused by water pollution. Therefore, some suggestions about strengthening water intervention management and carrying out intervention water quality measures have been put forward.

In addition, for health risk of waste management, Ruppen et al. reported that Hwange, Western Zimbabwe used a community-based monitoring to identify sources of pollution and related these to past and present mining activities in a river downstream of a coal mining area. The primary source of acid mine drainage came from abandoned underground mine sites. Concentrations of Mn, Ni, and As were exceeding national fresh water guidelines and international drinking water standards. Results showed that this community-based monitoring offers a promising approach to establish a high-quality dataset for assessing risks.

## Main findings and research needed

The exposure to bioaerosols in solid or liquid waste processing facilities and their surrounding can lead to different types of adverse effects. Although they are well-documented by epidemiological surveys and numerous studies have monitored the air microbial contamination in these settings, a clear link between the environmental microbial pollution and diseases of workers or population is still lacking.

To better understand the relations between bioaerosol concentration and composition and risks for human health future research is needed, addressing several points:

1) Epidemiological studies and dose-response estimation. The evaluation of relationship between microbial contamination and health problems is very complex, because of the diversity of microorganisms in the bioaerosol and the variability of their distribution according to the area and time, as well as the difference in human susceptibility and reactions. Large and long lasting cohort studies, monitoring the air microbial contamination during the follow up could provide answers, but they are hampered by the complexity and costs. Moreover, the current use of personal protective equipment would hide the real risk.
2) Exposure assessment. The choice of appropriate indicators or index pathogens should take in account the aim of the study and their measure should be easy, timely, and representative of risk. For safety purposes total bacterial and fungal counts and fecal indicators are commonly used. Nevertheless, the lack of epidemiological studies hampers the definition of limits for an acceptable risk. On the other hand, the study of microbial composition of bioaerosol with cultural or non-cultural methods can show the presence and concentration of pathogens, allowing a more specific risk assessment (5, 6). Nevertheless, the proposed methods are highly variable and often lacking validation and standardization for risk assessment purposes. Thus, further studies are needed taking in account sensitivity and specificity, besides the real meaning of analytical results, e.g., of non-cultural methods toward the pathogens infectivity.
3) Risk assessment. The application of the qualitative or semi-quantitative methods can be useful for risk management, to apply preventive and protective measures, but only the Quantitative Microbial Risk Assessment (QMRA) can allow defining acceptable limits and to simulate and evaluate different scenarios (7). Moreover, interactions between bioaerosol infective components and air particulate and gaseous pollutants should be better studied to clarify their role in enhancing or reducing the microbial risk.

## Author contributions

CY, CV, YH, and AC contributed to conception and design of the study, organized the database, performed the statistical analysis, wrote the first draft of the manuscript, wrote sections of the manuscript. All authors contributed to manuscript revision, read, and approved the submitted version.
